# Computed tomography and magnetic resonance imaging of congenital thoracic systemic venous anomalies

**DOI:** 10.1007/s00247-022-05570-w

**Published:** 2023-01-05

**Authors:** Nihal M. Batouty, Ahmed M. Tawfik, Donia M. Sobh, Ahmed A. K. A. Razek

**Affiliations:** Department of Diagnostic and Interventional Radiology, Mansoura University, Faculty of Medicine, 12 El-Gomhoreya St., Mansoura, 35112 Egypt

**Keywords:** Azygos vein, Brachiocephalic vein, Children, Computed tomography, Congenital heart disease, Inferior vena cava, Magnetic resonance imaging, Superior vena cava

## Abstract

We present the imaging findings of thoracic systemic venous anomalies diagnosed by computed tomography and magnetic resonance imaging. Persistent left superior vena cava is the commonest anomaly of the thoracic systemic veins encountered either incidentally as an isolated finding or associated with congenital heart disease. Inferior vena cava (IVC) interruption with azygos continuation is the second most common anomaly, which may also be isolated or be associated with left isomerism syndrome. The article will also discuss other rarer systemic venous anomalies including retroaortic brachiocephalic vein and IVC drainage into the left atrium. Finally, the impact of pre-procedure reporting of thoracic systemic venous anomalies on the choice of intervention and patient outcome will be addressed.

## Introduction

Anomalies of the systemic veins are generally rare in a healthy population, with an increased prevalence in congenital heart disease patients. The most common abnormalities encountered in clinical practice are persistent left superior vena cava (SVC) and inferior vena cava (IVC) interruption with azygos or hemiazygos continuation. There are other less frequent anomalies such as retroaortic brachiocephalic vein or even more rare anomalies such as IVC draining into the left atrium instead of normal drainage into the right atrium [1–3]. Systemic vein anomalies may be incidental findings detected on cross-sectional imaging in children or adults. Therefore, general and pediatric radiologists should be aware of such findings. Abnormalities in the systemic veins may affect the placement of venous cannulation and are of utmost importance before open heart surgery, directly affecting the choice of cardiopulmonary bypass performed [4].

## Embryology

**Fig. 1:**
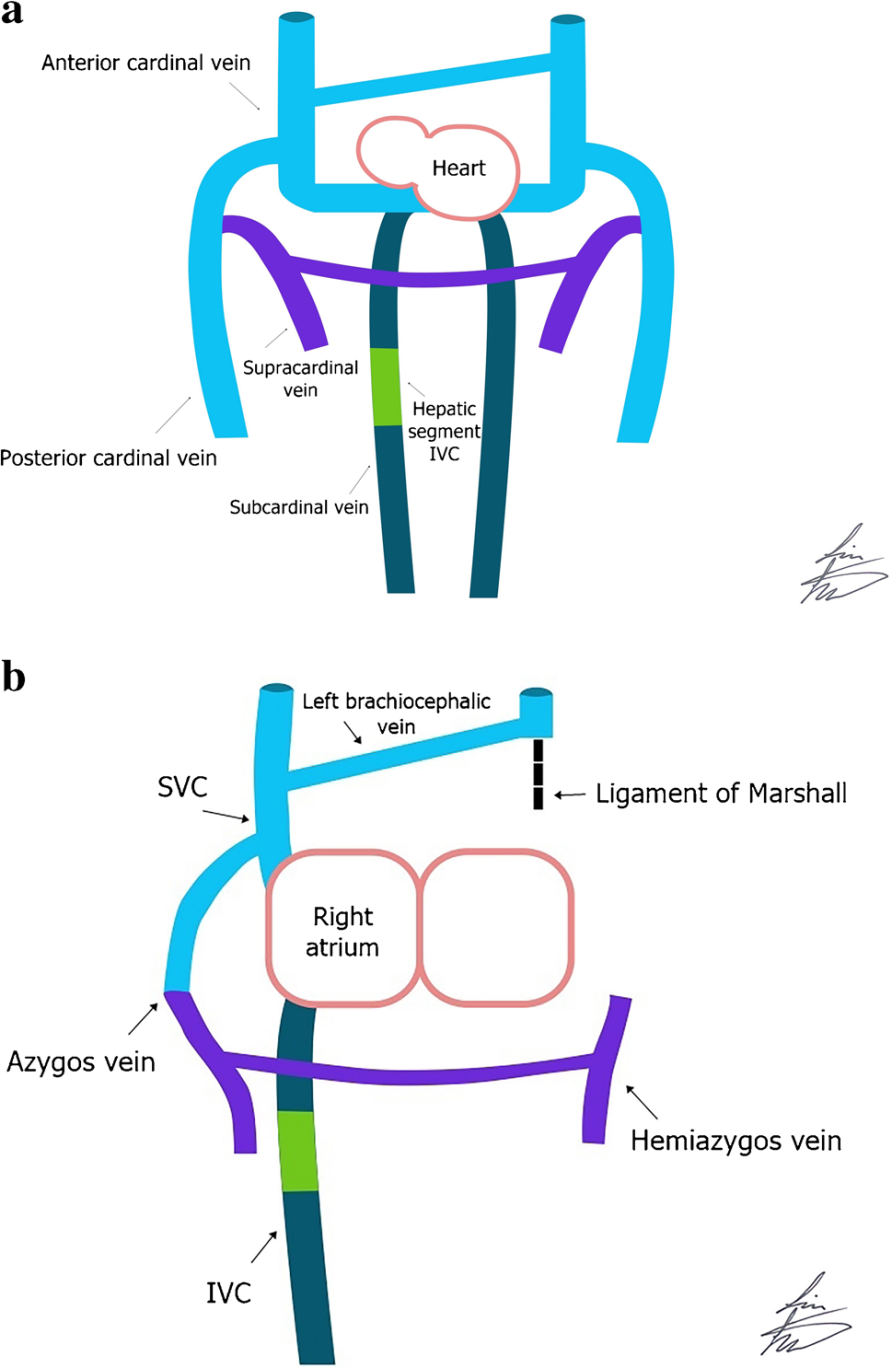
The illustration shows the embryology (**a**) and anatomical (**b**) development of the superior vena cava (SVC), the inferior vena cava (IVC), the left brachiocephalic vein and azygos and hemiazygos veins

By the beginning of the 4^th^ intrauterine week, the primitive vitelline–umbilical–cardinal vein system develops. The upper body is drained by bilateral anterior cardinal veins, whereas the distal body is drained by bilateral posterior cardinal veins. Figure 1 illustrates the embryological development and anatomy of the systemic veins. The paired anterior and posterior cardinal veins join to form the common cardinal veins that drain into the sinus venosus in the 5^th^ week of gestation [5–7]. The left and right anterior cardinal veins become connected by an anastomosis that subsequently forms the left brachiocephalic vein. The normal right SVC is formed from three different segments: part of the right anterior cardinal vein (proximal to the brachiocephalic anastomosis), the right common cardinal vein and the right horn of the sinus venosus, which may explain why SVC anomalies are the commonest [7–9]. The distal portions of both anterior cardinal veins become the right and left internal jugular veins. Normally, the proximal left anterior cardinal vein regresses and forms the ligament of Marshall. The coronary sinus develops from part of the left common cardinal vein and the left horn of the sinus venosus [2, 8, 9]. Persistent left SVC occurs when the proximal left anterior cardinal vein and the adjacent portion of the left common cardinal vein fail to regress. This embryological origin explains why right atrial drainage through the coronary sinus is the commonest site for drainage of persistent left SVC [8]. The cardinal veins that form the right SVC also share in the development of the azygos arch in addition to the proximal right posterior cardinal vein. While the proximal left posterior cardinal vein forms the great cardiac vein [7]. The caudal half of the embryo is drained not only through the posterior cardinal vein but also through the later developed paired supracardinal veins. The right supracardinal vein forms the azygos vein. The left supracardinal vein forms the hemiazygos and accessory hemiazygos veins [7–9]. The IVC results from fusion and anastomosis of multiple segments of vitelline and supracardinal, subcardinal and posterior cardinal veins, the complexity of which leads to a high likelihood of anomalies. IVC interruption or the absence of its suprarenal segment occurs due to failure of anastomoses of the right subcardinal vein to the liver, with IVC draining into the azygos or hemiazygos arch, and the hepatic veins draining into the right atrium [10].

## Computed tomography and magnetic resonance imaging techniques

Thoracic systemic venous anomalies may be suspected/diagnosed by different imaging modalities [11–19] including echocardiography [20–23] and best detected with computed tomography (CT) angiography [24–30] or magnetic resonance (MR) angiography [31–39].

### Computed tomography angiography

In our experience, retrospective low-dose electrocardiogram-gated cardiac CT 64-slice or higher multi-detector scanner is preferred for the imaging of congenital heart diseases in infants and young children (80 kVp, 150–200 mA, collimation 128 × 0.6 mm). Contrast medium (preferred concentration: 350 mg iodine/mL) is injected intravenously (dose = 0.5–2 mL/kg, rate = 1–2 mL/s). It is recommended to routinely acquire a delayed venous phase for better contrast opacification of the venous structures and detection of venous anomalies, regardless of the site of injection. The scan should extend from the origin of the aortic arch branches in the lower neck to the upper abdomen [11–13].

### Cardiac magnetic resonance imaging

Cardiac MRI is usually indicated in congenital heart diseases with systemic venous anomalies when there is a need for volumetric or functional assessment of ventricles or flow dynamics of the great vessels either pre- or postoperative, in addition to postoperative shunt evaluation. Cardiac MRI requires not less than a 1.5-tesla MRI machine. The MRI protocol at our institution includes routine cardiac cine steady-state free precession (SSFP) sequences in short axis, 2-chamber, 3-chamber and 4-chamber planes (repetition time [TR] 3.2–3.6 ms, echo time [TE] 1.6–1.8 ms, field of view [FOV] 350 mm^2^, slice thickness 8 mm, slice gap 1 mm). Axial and coronal cine steady-state free precession images of the thorax are acquired to depict venous anatomy and anomalies such as persistent left SVC or IVC interruption with azygos or hemiazygos continuation [11, 14]. Phase contrast sequences are acquired for flow quantification in major arterial and venous structures when needed. In addition, different MR angiography techniques are available for vascular imaging of the thorax. The 3-D balanced SSFP imaging sequence does not require contrast injection but is limited by a long acquisition time and susceptibility to artifacts. For contrast-enhanced MR angiography, time-resolved MR angiography is preferred over static contrast-enhanced MR angiography because it avoids the necessity of precise timing of image acquisition after contrast bolus and data are continuously acquired throughout the pass of contrast in vessels [3]. Lastly, advances such as 4-D flow imaging may be added and have the potential to provide additional hemodynamic information.

### Classification

Table 1 shows the classification of thoracic systemic venous anomalies and Table 2 shows different types of abnormal SVC drainage.

**Table 1:** Classification of thoracic systemic venous anomalies

Anomalies of superior vena cava	Persistent left superior vena cava Single left superior vena cava Low atrial insertion and abnormal drainage of right superior vena cava Congenital aneurysm of right superior vena cava
Inferior vena cava interruption and abnormal drainage	Inferior vena cava interruption with azygos/hemiazygos continuation Abnormal drainage of inferior vena cava
Anomalies of azygos system	Azygos lobe/hemiazygos lobe Absent azygos vein Abnormal drainage of azygous system
Anomalies of brachiocephalic vein	Retroaortic or subaortic brachiocephalic vein Double brachiocephalic vein Congenital aneurysm of brachocephalic vein

**Table 2:** Abnormal drainage of superior vena cava

Persistent left superior vena cava	Drainage into right atrium via coronary sinus Direct drainage into left atrium Drainage through accessory hemiazygos vein to azygos vein then right superior vena cava and right atrium
Single left superior vena cava	Mirror image of normal right superior vena cava (situs inversus totalis) Persistent left superior vena cava with absent right superior vena cava
Right superior vena cava	Drainage into left atrium

## Anomalies of the superior vena cava

### Persistent left superior vena cava (bilateral superior vena cava) (Fig. 2)

**Fig. 2:**
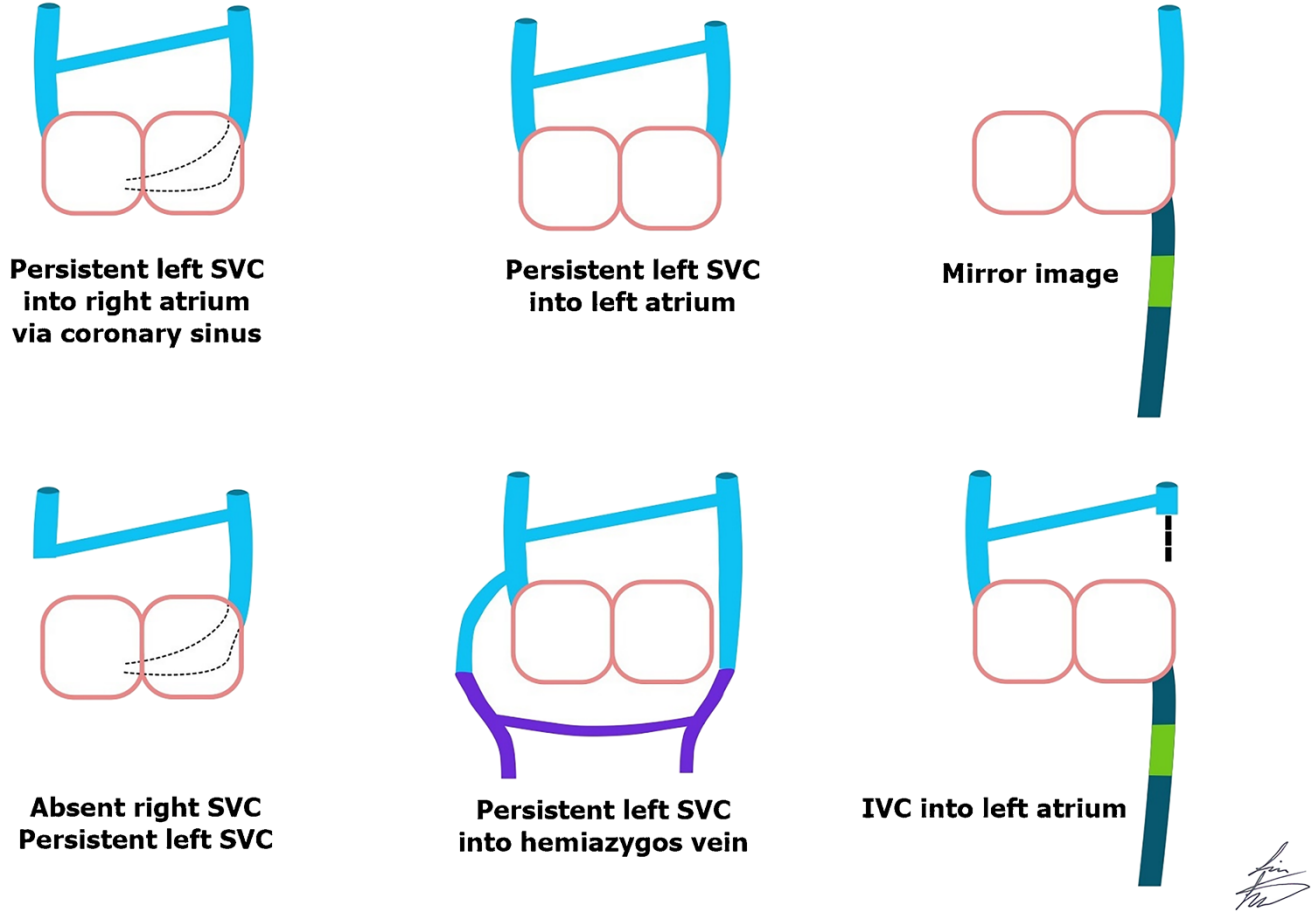
The illustration shows a persistent left superior vena cava (SVC) and its variations. The commonest drainage of a persistent left SVC is to the right atrium via the coronary sinus. The right lower corner image shows the inferior vena cava (IVC) draining into the left atrium

Persistent left SVC is the commonest thoracic systemic venous anomaly. It may be isolated in 0.5% of the normal population, being asymptomatic, and discovered incidentally on CT or MRI of the thorax. The reported prevalence of persistent left SVC in patients with congenital heart diseases is 8–10% [1, 2, 16]. Persistent left SVC commonly occurs in patients with chromosomal abnormalities such as Turner syndrome (13%), trisomy 18 (12%) and trisomy 21 (7%) [17, 18]. It may be associated with atrioventricular septal defects, conotruncal anomalies and aortic coarctation [19–21]. A persistent left SVC is more frequently associated with heterotaxy than with situs solitus or inversus, with a prevalence of 60–70%. Persistent left SVC is more common in right isomerism than left isomerism [3, 22]. The left internal jugular vein and left subclavian vein unite to form the left brachiocephalic vein. As a result of failure of left anterior cardinal vein regression, the left brachiocephalic vein continues as a persistent left SVC that descends on the left side of the mediastinum, lateral to the aortic arch and anterior to the left lung hilum. A persistent left SVC enters the pericardium to the left posterior atrioventricular groove, then passes along the ligament of Marshall to drain into the right atrium through the coronary sinus. This route of drainage is the most common and is usually asymptomatic because there is no blood shunt or hemodynamic change and therefore requires no surgery [11]. The coronary sinus ostium may be narrowed leading to difficult introduction of intravenous lines, pacemakers or defibrillator leads. Coronary sinus ostium atresia rarely occurs, but would nevertheless receive coronary venous return, followed by retrograde flow from the persistent left SVC to the left brachiocephalic vein. In such cases, ligation of the persistent left SVC during cardiac surgery leads to myocardial ischemia and acute coronary venous hypertension [16].

Bridging of the left brachiocephalic vein (connecting both the right SVC and the persistent left SVC) is reported in 27–32% of patients. When there is a bridging vein, the persistent left SVC is usually smaller than the right SVC [3, 19]. The presence and size of the bridging left brachiocephalic vein should be reported on CT and MRI studies [11].

On echocardiography, a dilated coronary sinus is the crucial key to the diagnosis of persistent left SVC. The coronary sinus may not be dilated if the persistent left SVC is small and the bridging vein is large; in this condition, echocardiography can miss the diagnosis. A dilated coronary sinus draining the persistent left SVC can cause arrhythmia because of A-V node stretching [23, 24].

#### Drainage into the right atrium through the coronary sinus

**Fig. 3:**
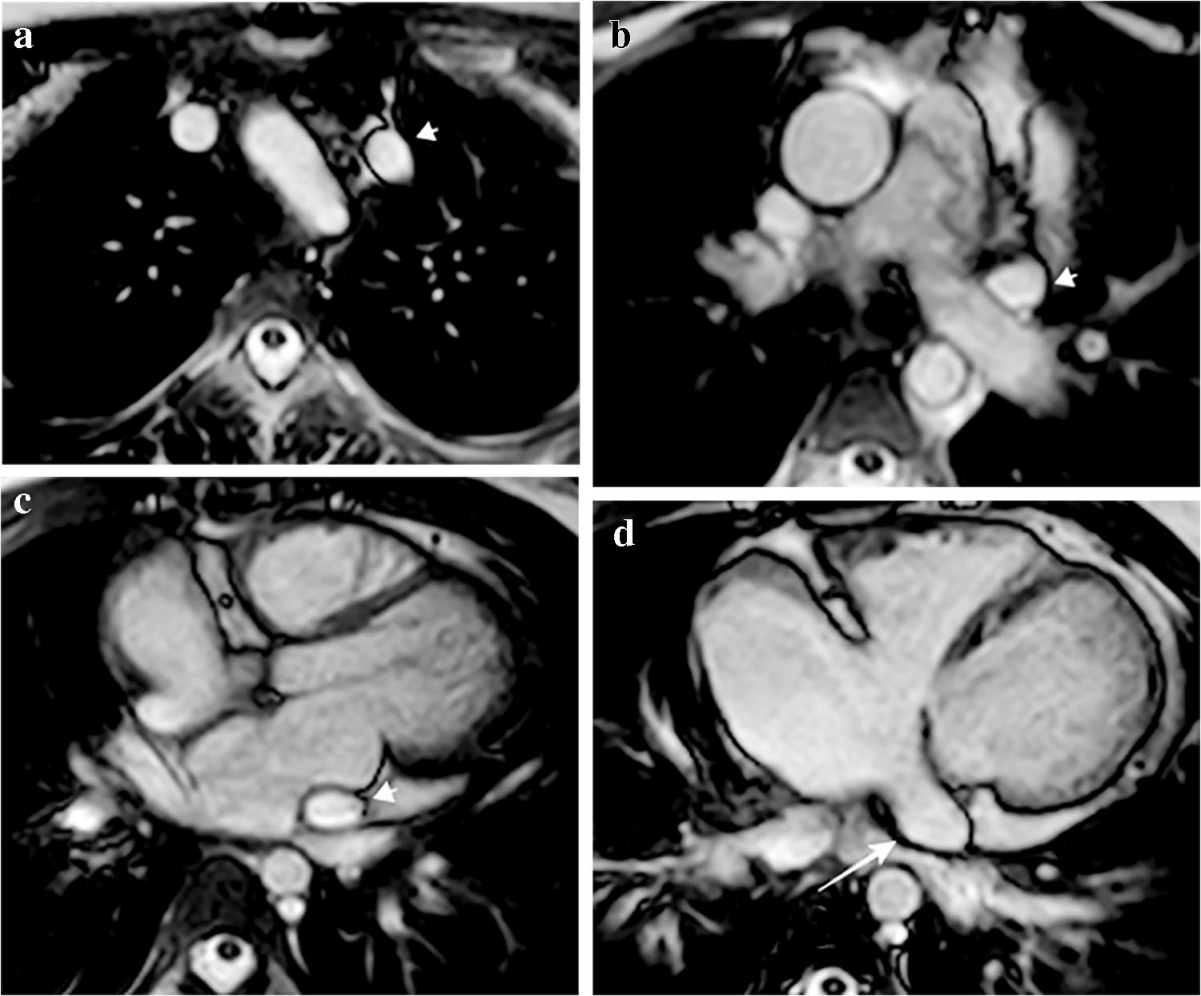
A persistent left superior vena cava (SVC) with no other anomalies in a 12-year-old girl. **a–d** Axial cine steady-state free precession cardiac magnetic resonance images show a persistent left SVC (*short arrow*) along its course on the left side of the mediastinum lateral to the aortic arch (**a**), anterior to the left hilum (**b**), in the left atrioventricular groove (**c**) and draining into the right atrium via the coronary sinus (**d***, long arrow*)

**Fig. 4:**
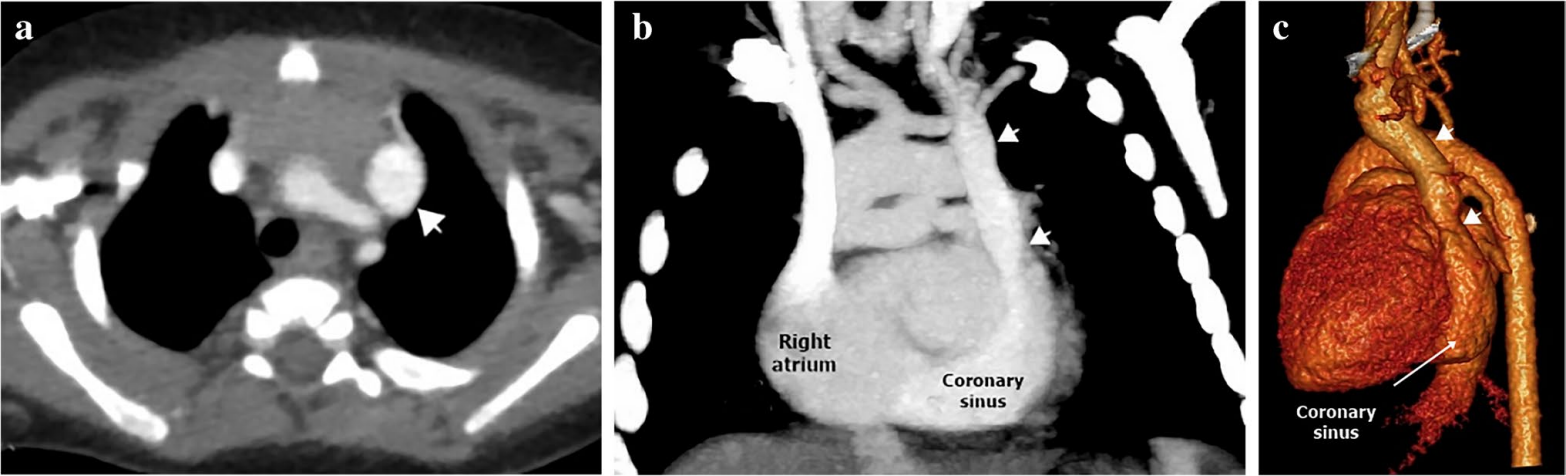
A persistent left superior vena cava (SVC) with no other anomalies in a 5-month-old boy. (**a**) An axial computed tomography (CT) image shows the persistent left SVC *(arrow)* on the left side of the mediastinum lateral to the aortic arch branches. **b**, **c** Coronal oblique CT image (**b**) and volume-rendered CT image (**c**) show drainage of the persistent left SVC *(arrows)* into the right atrium via the coronary sinus

In situs solitus, the persistent left SVC drains into the right atrium through the coronary sinus **(**Figs. 3 and 4).

#### Direct drainage into the left atrium

**Fig. 5:**
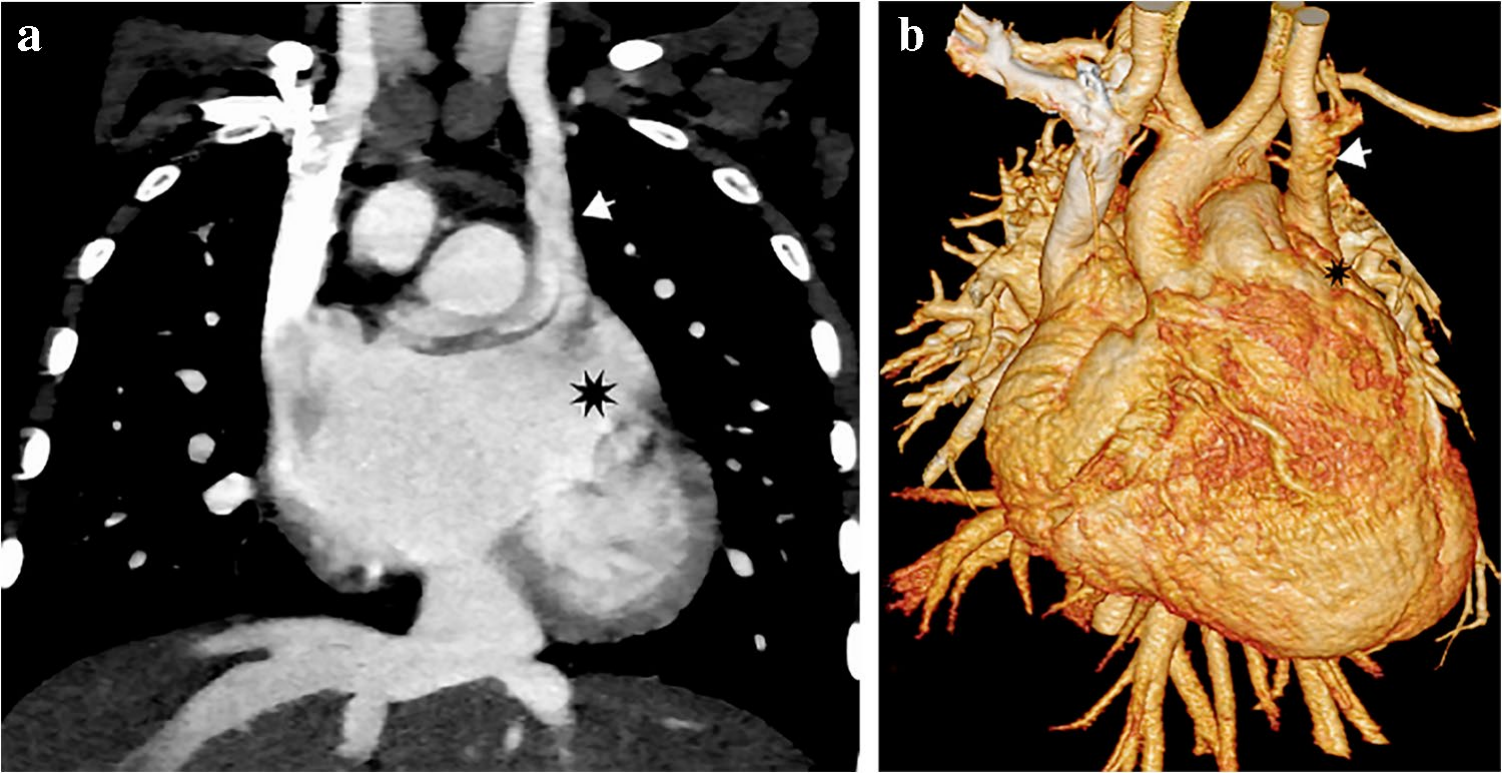
A persistent left superior vena cava (SVC) in a 2-year-old girl with left isomerism. **a, b **Coronal oblique computed tomography (CT) image (**a**) and volume-rendered CT image (**b**) of the persistent left SVC (arrows) show direct drainage into the left side of the common atrium (*asterisk*). The suprahepatic inferior vena cava drains into the posterior wall of the common atrium

**Fig. 6:**
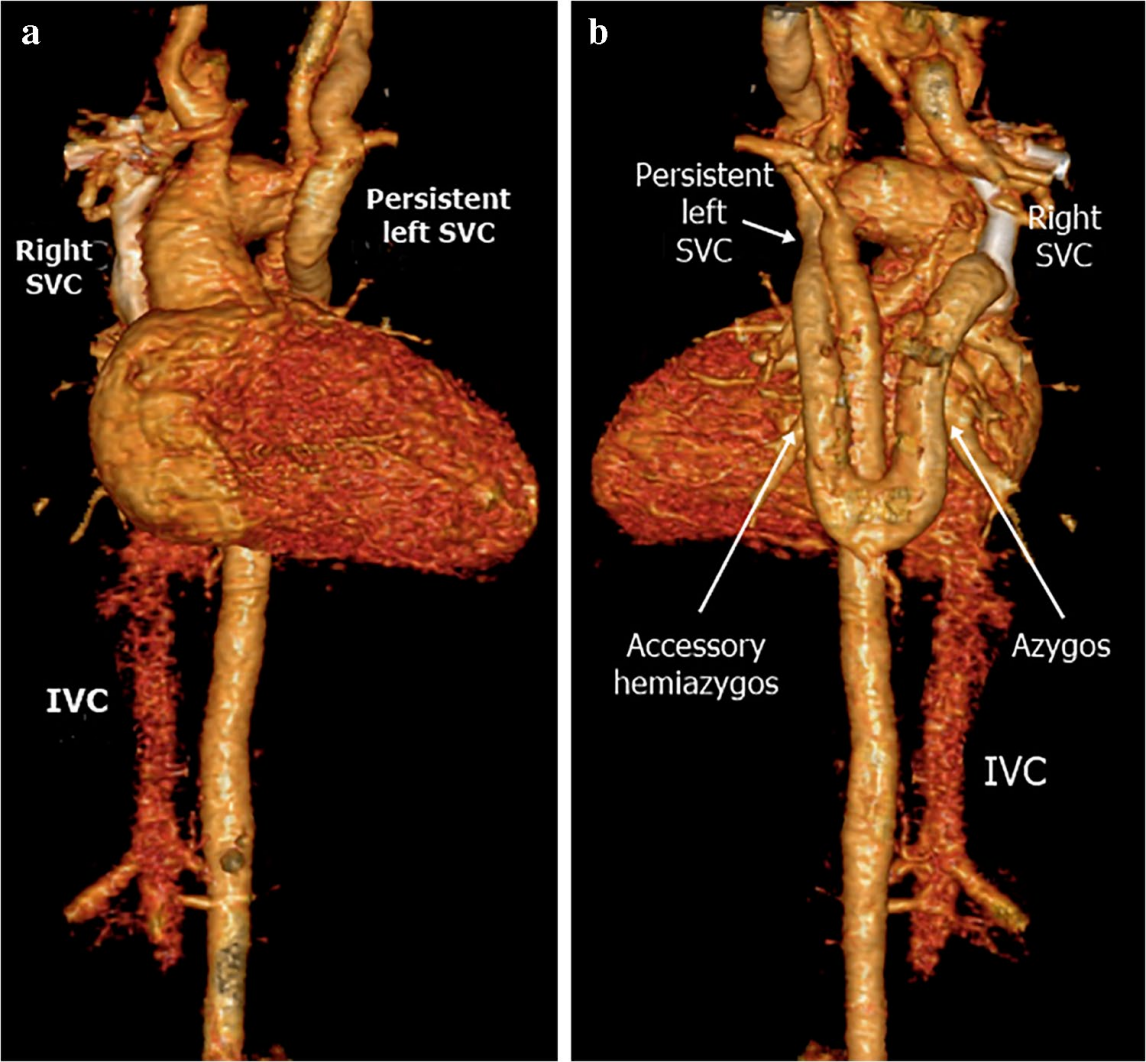
A persistent left superior vena cava (SVC) in a 1-year-old boy draining through an accessory hemiazygos vein to the azygos vein then the right SVC and right atrium. **a**, **b** There is no connection to the coronary sinus or the left atrium on anterior (**a**) or posterior (**b**) volume-rendered computed tomography images. Note the coarctation of aortic isthmus. *IVC* inferior vena cava

In heterotaxy, the persistent left SVC drains directly into the atrium. Being a left-side structure, the coronary sinus is often absent in right isomerism where there is direct drainage of the persistent left SVC into the left-side atrial roof [11, 22]. In left isomerism, the persistent left SVC drains into the right atrium through the coronary sinus in only 50%; otherwise, it has direct drainage into the left-side atrium [3] (Fig. 5). An extremely rare form of drainage of the persistent left SVC was described [25], in which the persistent left SVC drains through the accessory hemiazygos vein to the azygos vein, then to the right SVC and the right atrium, without any connection to the coronary sinus or left atrium **(**Fig. 6).

The surgical approach in pediatric patients with congenital heart diseases, such as cavopulmonary connection (Glenn shunts), is dependent on the bridging vein; if its size is adequate, a unilateral cavopulmonary connection is sufficient. If the bridging vein is absent, a bilateral procedure is mandatory [4].

**Fig. 7:**
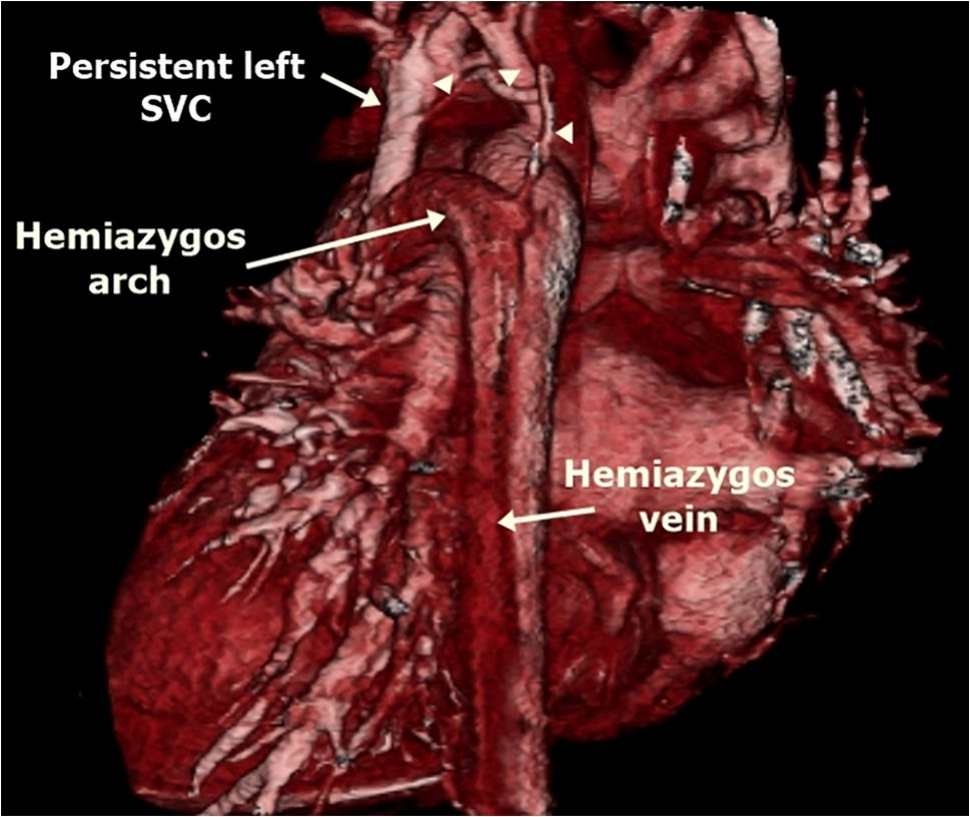
A left superior intercostal vein as a tributary of a persistent left superior vena cava (SVC) in a 4-month-old girl with left isomerism. A posterior volume-rendered computed tomography image shows the left superior intercostal vein (*arrowheads*) and hemiazygos continuation of the inferior vena cava interruption draining into the persistent left SVC

The left superior intercostal vein is a tributary of a persistent left SVC in 20% of patients (Fig. 7). It passes on the left side of the aortic arch to join the persistent left SVC or left brachiocephalic vein [3, 11]. It drains from the second to fourth left posterior intercostal veins. The left superior intercostal vein is connected to the accessory hemiazygos vein in 75%. The left superior intercostal vein becomes dilated when the SVC is occluded in conditions with volume overload, such as in congestive heart failure and when the left brachiocephalic vein is hypoplastic. A dilated left superior intercostal vein may be confused for persistent left SVC but can be distinguished by following its origin and drainage [2, 26].

Mimickers may be confused with persistent left SVC on cross-sectional imaging when there is a vessel on the left side of the aortic arch in the mediastinum. These mimickers include total or partial anomalous pulmonary venous drainage of the left upper pulmonary vein. Their differentiation from persistent left SVC can be achieved by tracking the origin inferiorly from the lung lobes [3, 11, 15, 27]. The size of the left brachiocephalic vein might be helpful, being more dilated with anomalous pulmonary venous drainage than with persistent left SVC because in the former, it receives part or all the pulmonary venous return [16]. Another persistent left SVC mimicker is persistent levoatrial cardinal vein, which is an anomalous vein that connects pulmonary to systemic circulation (left atrium/or pulmonary vein to SVC/or left brachiocephalic vein); it may be associated with hypoplastic left heart syndrome [28]. The levoatrial cardinal vein only resembles a persistent left SVC with direct left atrial drainage; a persistent left SVC draining into the right atrium via the coronary sinus or into the left atrium via an unroofed coronary sinus or atrial septal defect is easily recognized. A persistent left SVC is located anterior to the left pulmonary artery, while the levoatrial cardinal vein is posterior [15, 28, 29]. Another possible pitfall of persistent left SVC is the retroaortic brachiocephalic vein. The normal left brachiocephalic vein passes anterior to the aortic arch to join the right brachiocephalic vein, forming the right SVC. A retroaortic brachiocephalic vein descends in the mediastinum on the left side of the aortic arch, then passes behind the aortic arch or esophagus before joining the right brachiocephalic vein [11, 15, 16].

Pericardiophrenic veins represent the venous drainage of the pericardium and diaphragm. They drain into the internal thoracic vein, superior intercostal vein or left brachiocephalic vein and are located lateral to the heart and mediastinum. When SVC or IVC obstruction or portal hypertension to occurs, the dilated left pericardiophrenic vein has a similar course to a persistent left SVC. Superiorly, both veins are located on the left side of the mediastinum lateral to the aortic arch and connected to the left brachiocephalic vein. They can be distinguished by following their course inferiorly, whereas a persistent left SVC connects to the coronary sinus or directly into the left atrium, while left pericardiophrenic vein descends toward the diaphragm [30].

There are different possibilities of left to right blood shunting that might occur with persistent left SVC. Drainage of a persistent left SVC into the right atrium through the coronary sinus does not usually cause blood shunting. However, the rise in pressure may contribute to the development of an unroofed coronary sinus, where communication develops between the coronary sinus and the left atrium. The direction of the shunt is likely to be left to right, depending on atrial pressure; atrial septal defect of coronary sinus type may develop [3]. Right-to-left blood shunt occurs when there is atresia of the ostium of the coronary sinus. Direct drainage of a persistent left SVC into the left atrium causes right-to-left shunt. Right-to-left shunting may be asymptomatic or cause mild cyanosis or, rarely, paradoxical embolism [31]. Reversal of this shunt is possible in the presence of a large bridging vein. A persistent left SVC with left atrial drainage and symptomatic right-to-left shunt requires surgical correction. A persistent left SVC is disconnected from the left atrium and ligated if there is a sizable bridging vein; if absent or a small bridging vein, a persistent left SVC is connected to the right SVC or to the appendage of the right atrium [4].

### Single left superior vena cava

#### Mirror image of normal right superior vena cava (situs inversus totalis) (Fig. 2)

**Fig. 8:**
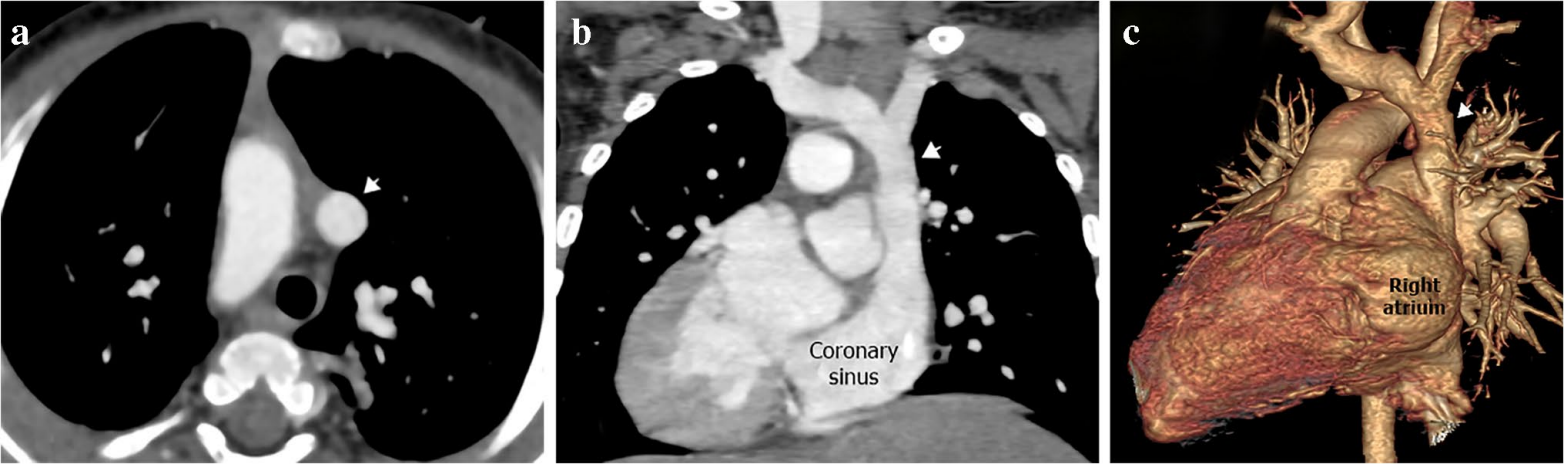
A single left superior vena cava (SVC) mirror image in a 3-year-old boy with situs inversus totalis. **a–c** Axial computed tomography (CT) image (**a**), coronal oblique CT image (**b**) and volume-rendered CT image (**c**) show a single left-side SVC (*arrows*) draining into the left-side right atrium

In situs inversus totalis a left-side SVC is the mirror image of the normal right-side SVC and there is no persistent left SVC. This condition is a merely a reversal of normal anatomy [11] (Fig. 8).

#### Persistent left superior vena cava with absent right superior vena cava (Fig. 2)

A persistent left SVC may be associated with an absent right SVC and has an incidence of 0.09–0.13%. It results from regression of the right anterior cardinal vein and persistence of the left anterior cardinal vein. The coronary sinus draining this isolated persistent left SVC may be markedly dilated because it receives all the venous return from the upper half of the body [24, 32].

### Abnormal drainage of right superior vena cava

**Fig. 9:**
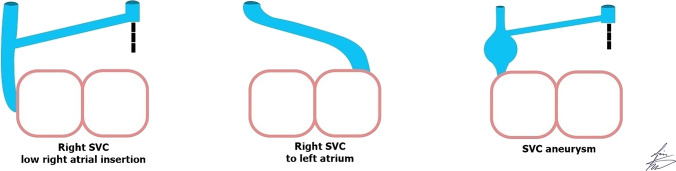
The illustration shows the right superior vena cava’s (SVC) low insertion into the right atrium, abnormal drainage into the left atrium and SVC aneurysm

Abnormal drainage of the right SVC includes abnormal low right atrial insertion of the right SVC into the right atrium or abnormal drainage of the right SVC into the left atrium (Fig. 9). The right SVC rarely drains into the left atrium without associated cardiac malformations [3, 16, 33].

### Congenital aneurysm of right superior vena cava

**Fig. 10:**
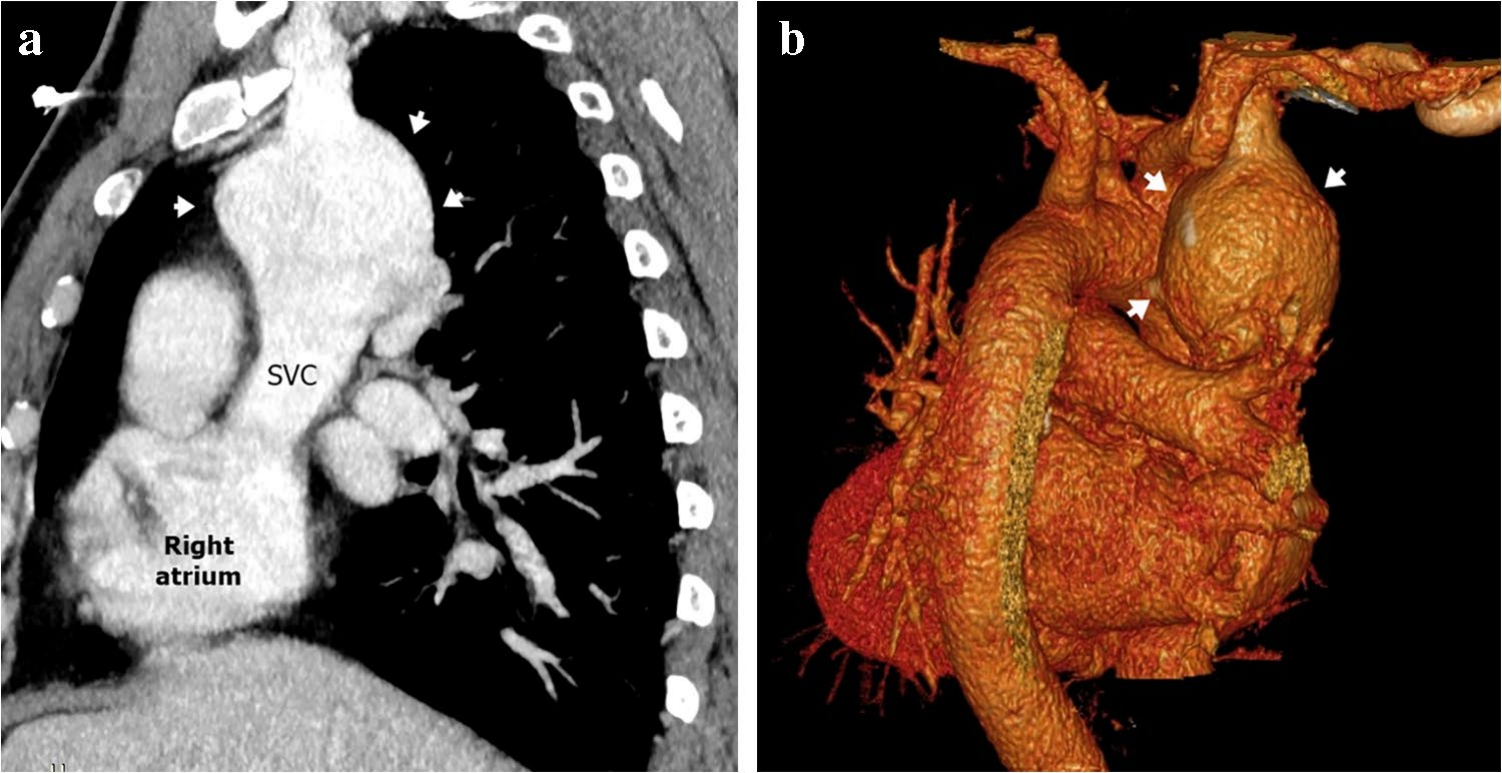
A right superior vena cava (SVC) aneurysm in a 62-year-old man. **a, b** Sagittal oblique computed tomography (CT) image (**a**) and volume-rendered CT image (**b**) show fusiform aneurysmal dilatation of the SVC (*arrows*)

Congenital aneurysm of the right SVC is an extremely rare abnormality, caused by congenital weakness of the SVC wall or absence of the longitudinal muscle layer. The aneurysm may be fusiform or saccular and while this lesion is asymptomatic and incidentally discovered, it has been associated with thrombosis and SVC obstruction [16, 34, 35] (Figs. 9 and 10).

## Inferior vena cava interruption and abnormal drainage

### Inferior vena cava with azygos/hemiazygos continuation

**Fig. 11:**
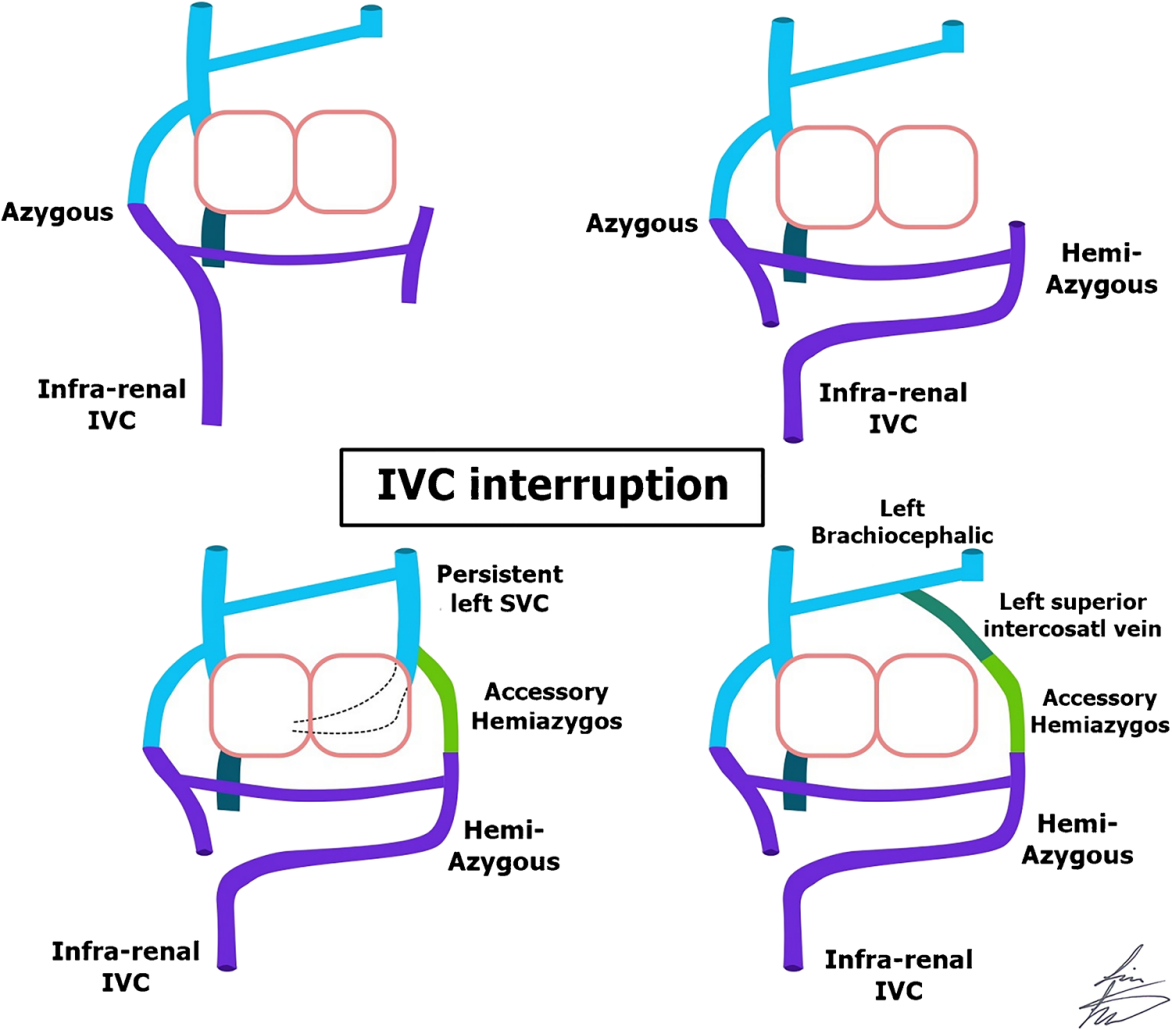
An illustration shows inferior vena cava (IVC) interruption and variations. Azygos continuation, then drainage into the right superior vena cava (SVC) via the azygos arch. Hemiazygos continuation and its three possible routes: (1) draining into the azygos vein, then to the right SVC; (2) draining into the accessory hemiazygos vein, then the persistent left SVC; and (3) draining into the accessory hemiazygos, left superior intercostal vein, left brachiocephalic vein, then to the right SVC

The IVC is composed of four anatomical segments: hepatic, suprarenal, renal and infrarenal. IVC interruption is considered the second most common systemic venous anomaly with a reported prevalence of up to 0.6%. It results from the absence or hypoplasia of the hepatic and suprarenal segments of the IVC due to atrophy of the right subcardinal vein. Blood flows from the infrarenal IVC through azygos and hemiazygos veins in the retrocrural and paraspinal areas to drain into the SVC (Fig. 11). A dilated azygos or hemiazygos vein is a predominant feature of this anomaly, as well as a dilated SVC. Hepatic veins drain into the right atrium via the suprarenal IVC [3, 36, 37]. IVC interruption with azygos or hemiazygos continuation is a near-constant feature of left isomerism but may be associated with other types of abnormal situs and congenital heart disease. It may be incidentally seen on imaging as an isolated finding in an individual with a structurally normal heart and normal situs [36].

**Fig. 12:**
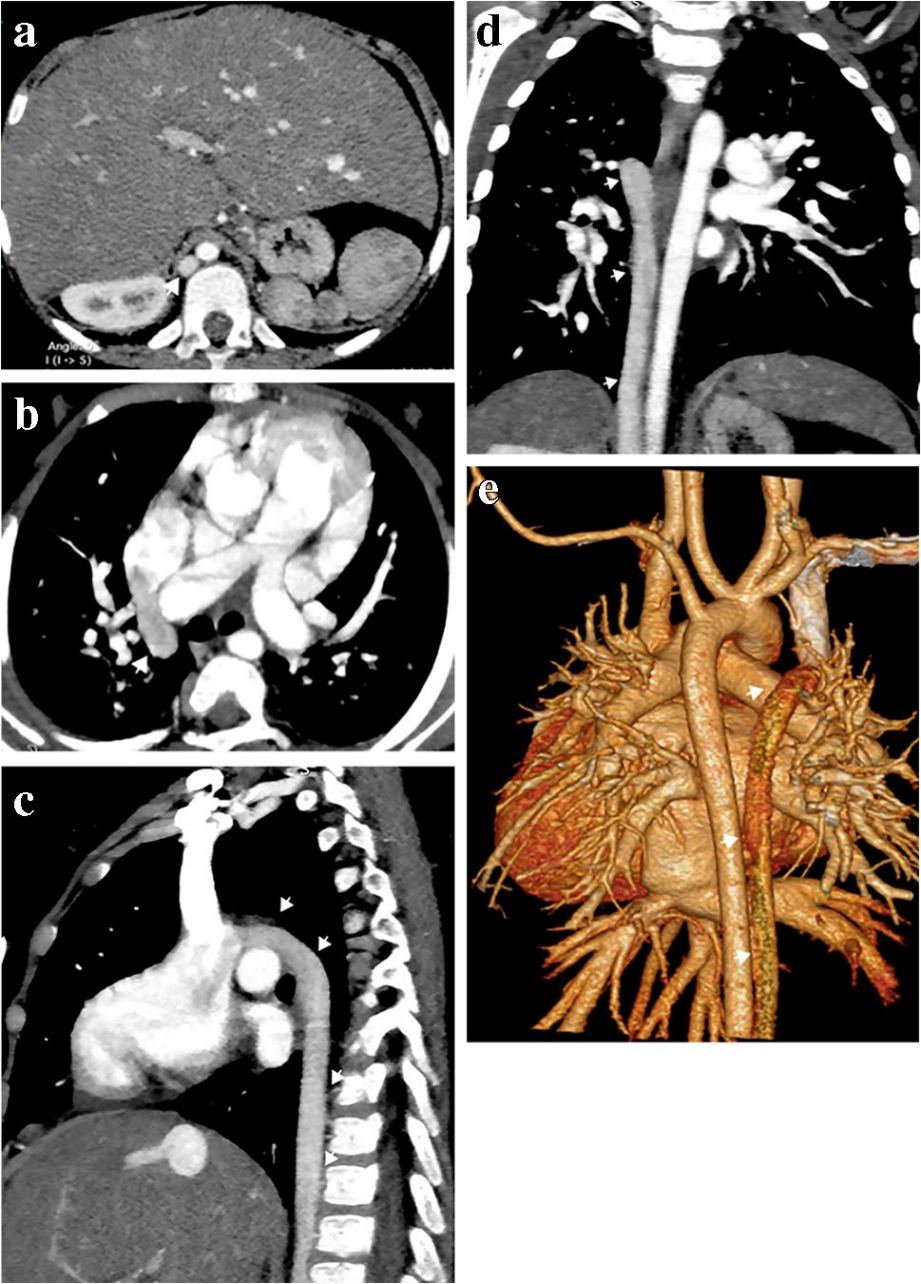
Inferior vena cava (IVC) interruption with azygos continuation in a 9-month-old boy with left isomerism. **a–e** Axial (**a, b**), sagittal (**c**) and coronal oblique (**d**) computed tomography (CT) images and volume-rendered CT image (**e**) show IVC interruption with azygos continuation (*arrows*) draining through the azygos arch into the superior vena cava (SVC). Note the polysplenia on the left side and central liver position

**Fig. 13:**
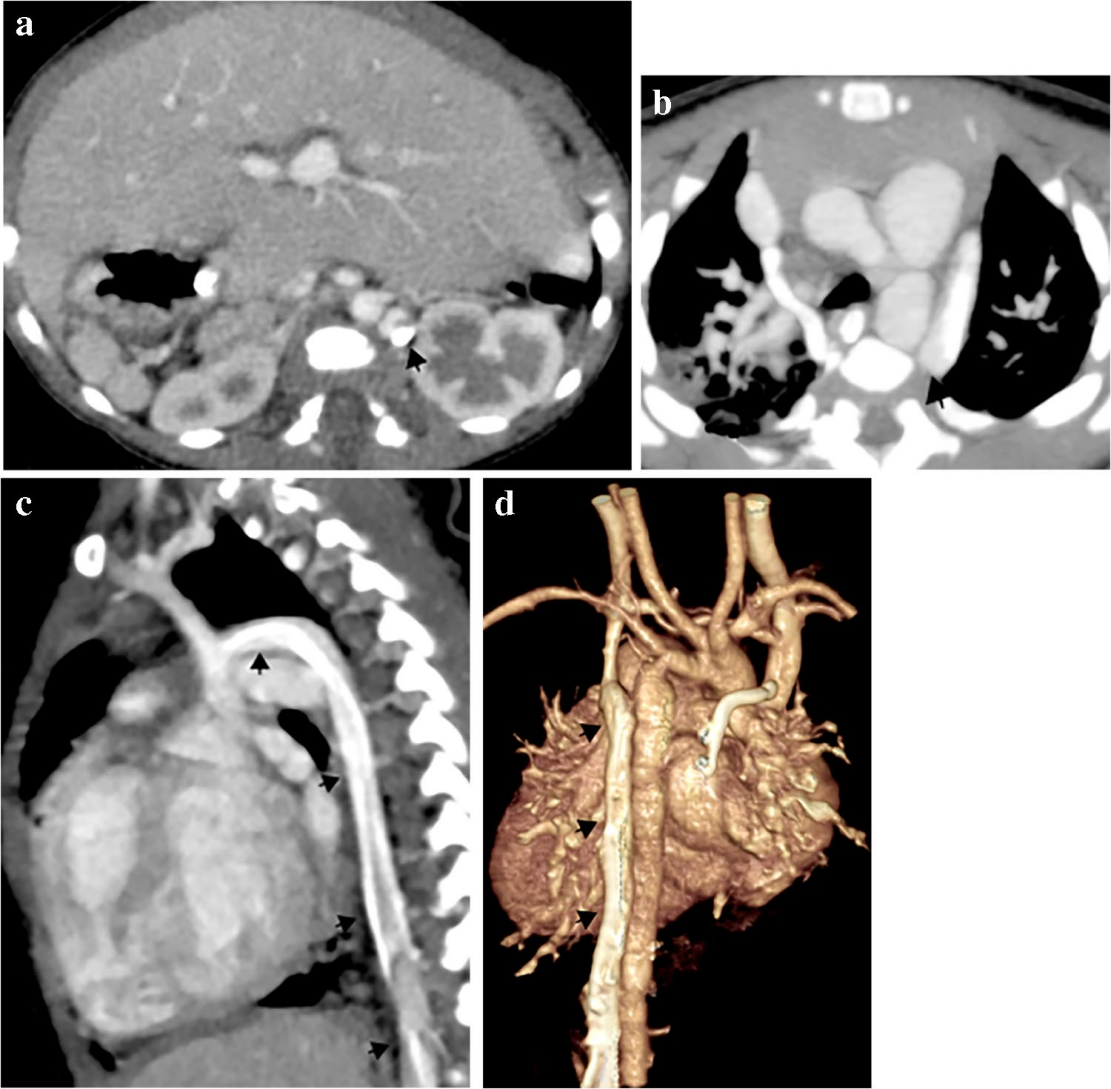
Inferior vena cava (IVC) interruption with hemiazygos continuation in a 5-month-old girl with left isomerism. **a–d** Axial (**a, b**), sagittal oblique (**c**) and volume-rendered (**d**) computed tomography (CT) images show IVC interruption with hemiazygos continuation draining into the persistent left superior vena cava (SVC) (*arrows*). Note the azygos arch draining into the right SVC, polysplenia on the right side and central liver position. The aortic arch is hypoplastic and there is aortic coarctation

Inferior vena cava interruption with azygos continuation is more frequent than with hemiazygos continuation. The azygos vein carries blood from the infrarenal IVC and drains into the right SVC via the azygos arch (Fig. 12). In the case of hemiazygos continuation, there are three possible routes for blood drainage (Fig. 11): (1) azygos vein to the right SVC; (2) accessory hemiazygos vein, then to a persistent left SVC (Fig. 13); and (3) accessory hemiazygos vein, left superior intercostal vein, left brachiocephalic vein, then to the right SVC [16, 37]. Associated congenital anomalies of abdominal IVC such as left-side IVC or double IVC can occur [36].

Presurgical reporting of IVC interruption is mandatory if the patient is undergoing a total cavopulmonary connection (modified Fontan surgery). The best surgical procedure is connecting the SVC (that drains the azygos continuation and thus the whole systemic venous return excluding the hepatic veins) to the right pulmonary artery. Radiologists must be aware of these surgical procedures for proper postoperative reporting, especially if complications develop such as venovenous collaterals in the mediastinum and paraspinal regions [3, 4, 11].

### Abnormal drainage of inferior vena cava

Normally, the IVC is a right-side structure that drains into the right atrium. There are two possibilities of left-side IVC: (1) situs inversus totalis, where there is a mirror image condition of the normal anatomy and the left-side IVC drains into the left-side morphological right atrium; and (2) complex congenital heart disease and heterotaxy, where the IVC may be left-side and drain into the left-side atrium (Fig. 2) or into the left side of a common atrium. Separate drainage of one or more hepatic veins into the right atrium has been reported. Rarely, IVC or hepatic veins can have an anomalous connection to the coronary sinus [3].

## Anomalies of the azygous system

### Azygos lobe/hemiazygos lobe

An azygos lobe implies the anomalous course of the azygos vein into the right lung apex in 0.4–1% of the population [16]. The abnormal position of the distal part of the azygos vein occurs due to the failure of the right posterior cardinal vein to migrate over the lung apex; instead, it penetrates it, pushing along overlying layers of visceral and parietal pleura [4–7]. This results in a mesoazygos, a mesentery-like structure containing the azygos vein [38, 39]. The azygos lobe is an asymptomatic anatomical variant that is easily diagnosed on imaging. A left azygos or hemiazygos lobe is rare and much less frequent than a (right) azygos lobe. It is caused by malposition of the left superior intercostal vein draining into the left brachiocephalic vein [3, 16].

### Absent azygos vein

Congenital absence of the azygos vein is usually asymptomatic. It is a very rare anomaly that results from the failure of the right supracardinal vein to develop. Azygos vein tributaries (right and left intercostal veins) will drain into the hemiazygos, accessory hemiazygos, left brachiocephalic and left superior intercostal veins, causing their dilatation [3, 16].

### Abnormal azygos vein drainage

Rarely, the azygos vein drains into the right brachiocephalic vein, right subclavian vein, intrapericardial SVC or right atrium [3].

## Anomalies of brachiocephalic vein

### Retroaortic or subaortic brachiocephalic vein

**Fig. 14:**
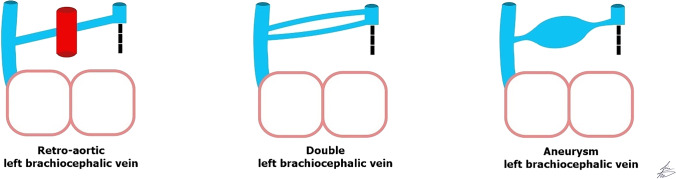
An illustration shows retroaortic brachiocephalic vein, double brachiocephalic vein and brachiocephalic vein aneurysm

**Fig. 15:**
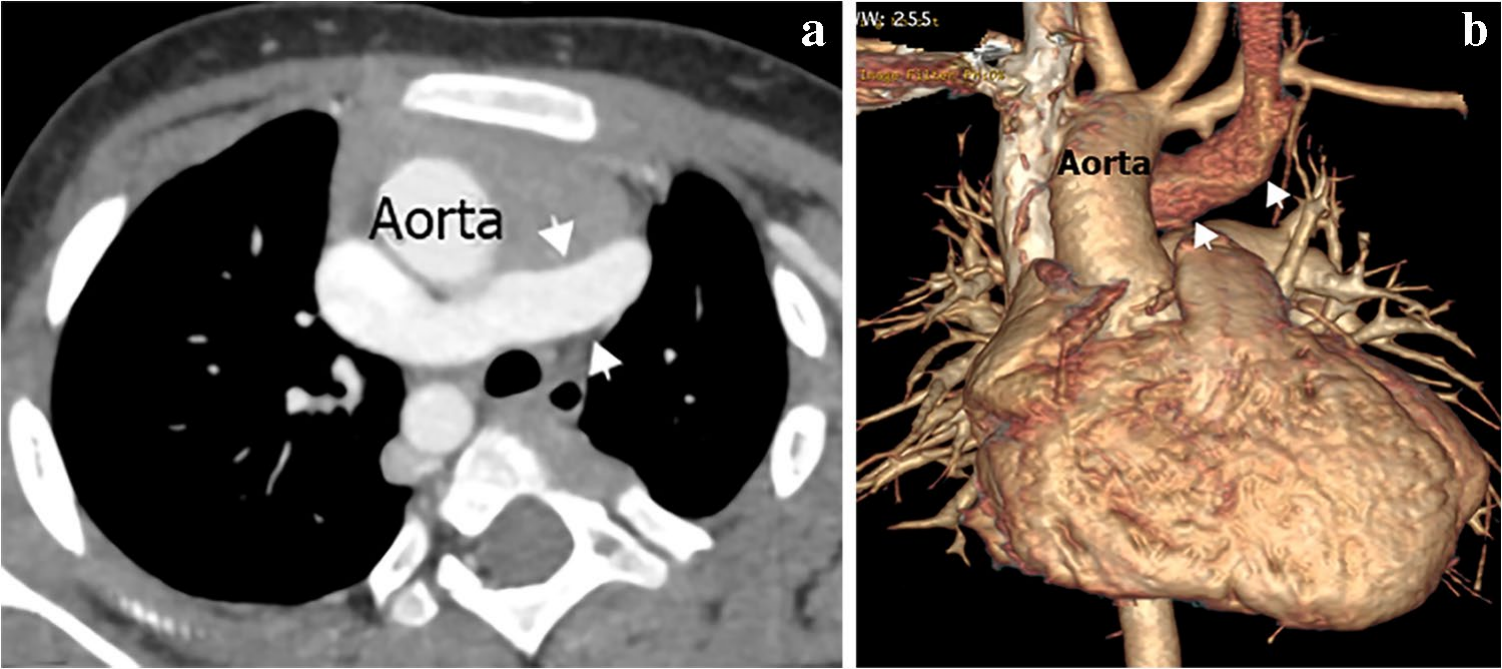
A retroaortic brachiocephalic vein with no other anomalies in a 3-year-old boy. **a, b** Axial (**a**) and volume-rendered (**b**) computed tomography images show retroaortic brachiocephalic vein (*arrows*)

**Fig. 16:**
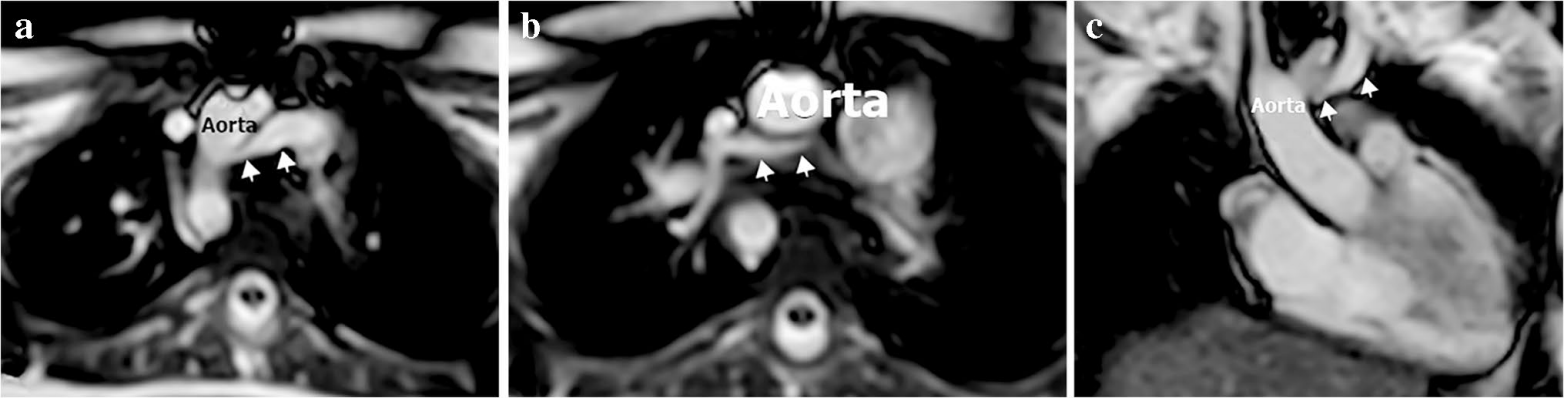
A retroaortic brachiocephalic vein with no other anomalies in 1.5-year-old boy. **a–c** Axial (**a, b**) and coronal (**c**) cardiac magnetic resonance cine steady-state free precession images show the retroaortic brachiocephalic vein (*arrows*)

Normally, the left brachiocephalic vein is formed from the left internal jugular and left subclavian veins. It courses obliquely and downward toward the right aspect of the upper mediastinum and passes anteriorly to the aortic arch and its branches. An anomalous left brachiocephalic vein is uncommon, representing 0.2–1% of congenital heart diseases [40]. The embryological cause of the anomalous brachiocephalic vein is unclear and may be related to the abnormal regression of the anastomosis between both anterior cardinal veins. A plexus of transverse precardinal anastomoses predisposes to different patterns of anomalous brachiocephalic vein: retroaortic, (circumaortic or subaortic) brachiocephalic vein, hypoplastic or absent brachiocephalic vein with bilateral SVC and double brachiocephalic vein [3]. When the left brachiocephalic vein courses inferiorly along the left side of the mediastinum and passes behind the ascending aorta or beneath the aortic arch to join the right SVC below the orifice of the azygos vein, it is called retroaortic or subaortic (Figs. 14, 15, 16). More rarely, it may have a retroesophageal or retrotracheal course [15, 40–43]. The left brachiocephalic vein may be absent or hypoplastic in the presence of a persistent left SVC. The venous return is drained via the left superior intercostal vein when the left brachiocephalic vein is absent. Reporting an anomalous brachiocephalic vein is crucial to avoid misinterpretation and the technical difficulty of inserting central venous catheters and cardiac interventions [3].

### Double brachiocephalic vein

**Fig. 17:**
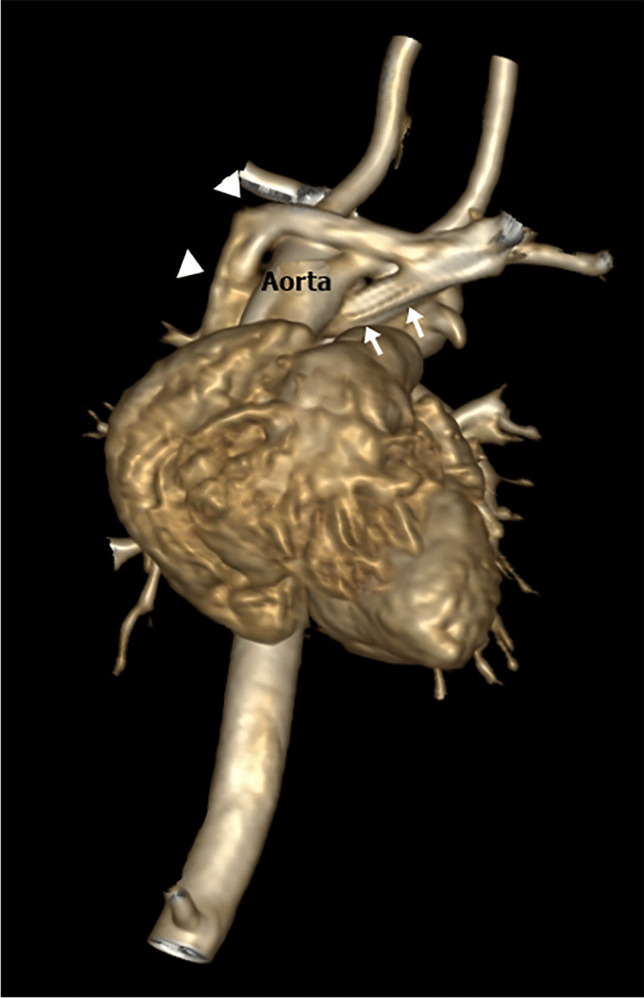
A double left brachiocephalic vein with no other anomalies in a 1-week-old girl. A volume-rendered computed tomography image shows one left brachiocephalic vein (*arrowheads*) passing anteriorly to the aorta and the other left brachiocephalic vein (*arrows*) passing posterior to the aorta

Double brachiocephalic vein is a rare anomaly with only a few cases reported worldwide. The theory of double brachiocephalic vein is the failure of both ventral and dorsal precardinal anastomosis to regress. Both brachiocephalic veins originate together, usually one brachiocephalic vein passes in its normal anatomical course in front of the aortic arch branches to join the right SVC. The other brachiocephalic vein might pass behind or inferior to the aortic arch before uniting with the right brachiocephalic vein [44–46] (Figs. 14 and 17).

### Congenital aneurysm of brachiocephalic vein

Congenital aneurysmal dilatation of the brachiocephalic vein is a rare anomaly, mostly asymptomatic and incidentally detected on imaging. The aneurysm may change its appearance according to patient positioning and is barely seen with the patient erect. Being an aneurysm, it may be complicated by thrombosis, venous occlusion or pulmonary embolism [3].

## Main points

- A persistent left SVC is the most common type of systemic venous anomaly encountered in daily clinical practice, either incidentally detected as a solitary finding or associated with congenital heart disease.
- IVC interruption with azygos continuation is the second most common anomayly; it may also be isolated or occur in association with polysplenia syndrome.
- Pre-procedure reporting of thoracic systemic venous anomalies impacts intervention and patient outcome.

## Data Availability

Data is available on reasonable request.
